# Picric Acid as a Test for Albumen

**Published:** 1884-06

**Authors:** 


### Picric Acid as a Test for Albumen.

The use of a saturated solution of picric acid as a test for albumen in the urine is a method which has recently been proposed; and although at first accepted as infallible by reason of its extreme delicacy, later investigations have shown that there are exceptions, and that it cannot always be depended upon. Some of these exceptions may be noted briefly as follows:

1st. If a patient whose urine is to be examined has been under the influence of quinine during the twenty-four hours preceding, the presence of a small quantity picric acid will produce at once a copious opalescent ring, which, on the application of heat, will be cleared up; but the application of heat or nitric acid will not produce coagulum. This shows, then, that the alkaloid quinine is eliminated through the kidneys, and the further proof, if any be needed, is found in the fact that picric acid, when placed in a solution of quinine, will produce the same effect as it does when the quinine is supposed to be in the urine.

2d. The presence of peptones in the urine, when the picric acid is added, will cause a precipitate. It has been shown by Gerhardt that peptones frequently appear in urine free of albumen, either as a forerunner or consequence of ordinary albuminuria, and Senator declares that peptones in slight quantities exist in every albuminous urine.

3d. An excess of albumen dissolves the precipitate formed by picric acid, and thus the examiner may be misled. It is therefore necessary to agitate the contents of the test-tube when a coagulum is formed by the addition of picric acid, in order to determine whether or not it will be dissolved.

4th. The presence of potassium salts in the urine will cause a precipitate.

5th. Still another possible cause of error is the presence in the urine of an excess of urates; but when this is the case, the reaction does not occur for some minutes. In regard to the latter, it should be noted that there is a difference between this reaction and that which occurs when albumen is present; that of the former, when closely examined, will be found to be made up of small crystals, which disappear on the application of heat, while the albumen reaction is granular, and, as a matter of course, is increased by the application of heat.

There is no question but that the use of picric acid is an exceedingly delicate test, but unless the possibilities of error are first eliminated, it will certainly prove, in many instances, misleading. Used as a means of confirming the diagnostic *after* the determination of the reaction, and the presence of the albumen by heat and nitric acid, it may prove valuable, but as a decisive test it is uncertain.—*Medical Bulletin.*

